# Randomized controlled phase III trial of adjuvant chemoimmunotherapy with activated cytotoxic T cells and dendritic cells from regional lymph nodes of patients with lung cancer

**DOI:** 10.1007/s00262-018-2180-6

**Published:** 2018-05-31

**Authors:** Hideki Kimura, Yukiko Matsui, Aki Ishikawa, Takahiro Nakajima, Toshihiko Iizasa

**Affiliations:** 1 Department of Thoracic Surgery, Saiseikai-Narashino Hospital, Izumi-cho 1-1-1, Narashino, Chiba 275-8580 Japan; 2 Department of Thoracic Surgery, Chiba Cancer Center, Chiba, Japan; 3 Department of General Thoracic Surgery, Graduate School of Medicine, Chiba University, Chiba, Japan

**Keywords:** Adjuvant therapy, Lung cancer, Regional lymph nodes, Cellular immunotherapy, Cytotoxic T cells

## Abstract

Randomized controlled trial of adjuvant chemoimmunotherapy for lung cancer indicated a significant advantage in patients receiving immunotherapy. Herein we report the final results and immunological analysis with a median follow-up of 59.6 months. Patients with post-surgical lung cancer were randomly designated to receive either chemoimmunotherapy (group A, immunotherapy arm) or chemotherapy (group B, control arm). The immunotherapy comprised the adoptive transfer of autologous activated killer T cells and dendritic cells (AKT–DC). The 2- and 5-year overall survival (OS) rates were 96.0 and 69.4% in group A and 64.7 and 45.1% in group B, respectively. Multivariate analysis results revealed that the hazard ratio was 0.439. The 2- and 5-year recurrence-free survival rates were 70.0 and 57.9% in group A and 43.1 and 31.4% in group B, respectively. Subgroup analysis for the OS between treatment groups indicated that younger patients (≤ 55 years: HR 0.098), males (HR 0.474), patients with adenocarcinoma (HR 0.479), patients with stage III cancer (HR 0.399), and those who did not receive preoperative chemotherapy (HR 0.483) had lower HRs than those in the other groups. Immunological analysis of cell surface markers in regional lymph nodes of subjects receiving immunotherapy indicated that the CD8^+^/CD4^+^ T-cell ratio was elevated in survivors. Patients with non-small-cell lung cancer benefited from adoptive cellular immunotherapy as an adjuvant to surgery. Patients with stage III cancer, those with adenocarcinoma, and those not receiving preoperative chemotherapy were good candidates. Lastly, cytotoxic T cells were important for a favorable chemoimmunotherapy outcome.

## Introduction

Progress in diagnostic procedures and surgical technology has considerably improved the prognosis of lung cancer surgery [1, 2]. In advanced cases, patient outcomes remain poor despite progress in adjuvant chemotherapy [3, 4] and molecular-targeted therapy [5]. Patients with stage IIIB and IV lung cancer and malignant pleural effusion, micrometastasis to mediastinal lymph nodes, or intrapulmonary metastasis are often identified after thoracotomy, shortly recur after surgery, and die early. Previously, we recruited advanced lung cancer patients with poor prognoses who had undergone surgery for improving prognosis by immunotherapy in combination with adjuvant chemotherapy or molecular-targeted therapy [6]. We present the results with a median follow-up of 59.6 months and the associated statistical immunological analyses.

## Patients and methods

The patients and methods are described in our previous report [6].

### Study design and inclusion criteria

Patients with post-surgical NSCLC were randomly assigned to receive either adjuvant chemoimmunotherapy (group A, immunotherapy arm) or adjuvant chemotherapy (group B, control arm). Immunotherapy consisted of the adoptive transfer of activated cytotoxic killer T cells and dendritic cells (AKT–DC) derived from the regional lymph nodes of patients with lung cancer. The patient inclusion criteria of this study were as follows: post-surgical patients, < 76 years; Eastern Cooperative Oncology Group performance status (PS), 0 or 1; adequate bone marrow, liver, and renal function; histology, primary NSCLC, including combined-type small-cell carcinoma; pathological stage, IB with a tumor size > 5 cm or with severe vessel invasion and II–IV (TNM staging system version 6). Patients with clinical stage I and II cancer received surgery and were pathologically stratified as group a: stage IB, group b: stage II, group c: stage IIIA, and group d: stage IIIB, IV. Patients with clinical stage IIIA cancer (single station N2 or T3N1) received two courses of induction chemotherapy and were stratified as group e: stage IIIA and group f: stage IIIB, IV diagnosed after thoracotomy (pathological stages). Patients with stages IIIB or IV cancer and malignant pleural effusion, micrometastasis to mediastinal lymph nodes, or intrapulmonary metastasis identified after thoracotomy were also included. Patients who underwent non-curative resection were included, but those with exploratory thoracotomies or macroscopic residual tumors were excluded from this study.

### Chemotherapy

We used platinum doublet regimens belonging to third-generation drugs as induction and adjuvant chemotherapy. Both groups received four courses of adjuvant chemotherapy after surgery (groups a, b, c, and d). Patients with clinical stage IIIA cancer (groups e and f) received two courses of induction and adjuvant chemotherapy. After the confirmation of recurrence, chemotherapy was resumed and EGFR-mutation-positive patients received EGFR-TKI. Immunotherapy was continued or resumed with the patient’s consent in combination with chemotherapy.

### Preparation of activated killer T cells and dendritic cells from regional lymph nodes

The procedure involved in the preparation of AKT–DC has been previously described [6]. One to two grams of tumor-draining lymph nodes (TDLN) from intrapulmonary to mediastinal lymph nodes with no metastasis was transferred to a sterile Petri dish and aseptically minced into 1-mm^3^ tissue fragments. The tissue preparation was then suspended in 50 ml Alyse (ALyS505N: Cell Science and Technology Institute, Inc., Sendai, Japan) serum-free lymphocyte medium containing 400 IU/ml human recombinant interleukin 2, transferred to a 75-cm^2^ culture flask, and incubated at 37 °C in air containing 5% CO_2_. When the TDLN started to release AKT–DC, the tissues and cells were transferred to culture bags. The AKT–DC were separated from the TDLN tissue by filtering through a nylon mesh and were then transferred to another set of bag, split 2–3 times, and harvested. Cells were suspended in the cryoprotective agent CP-1 (Kyokuto Pharm. Co., Tokyo, Japan) with 4% human albumin and stored at 5–10 × 10^9^ cells/bag (freeze bag F-100A: NIPRO Osaka, Japan) in − 80 °C until used. AKT–DC were intravenously infused 1 week after each course of chemotherapy and were then continued once a month for the first 6 months after resection and then every 2 months until 2 years after surgery.

### Immunological analysis

We selected patients who died within 3 years of recurrence (*n* = 7) and compared the cell surface markers with that of other patients (*n* = 42) who were alive at 3 years in group A. Mononuclear cells obtained from regional lymph nodes of the patients after surgery were stained with immunofluorescence and analyzed using flow cytometer before and 1–2 months after the initiation of in vitro culture in IL2 when the cells actively proliferated.

### Flow cytometer

Cells were labeled with human monoclonal anti-CD8, HLA-DR, CD80, and CD4 antibodies conjugated with fluorescein isothiocyanate (FITC; Becton, Dickinson and Co., NJ, USA) and anti-CD3, B7H1 (PD-L1), CD83, and CD25 antibodies conjugated with phycoerythrin (PE) and counted using a flow cytometer (Cytomics-FC500; Beckman Coulter, CA, USA). 7-Amino-actinomycin D (7AAD) was added to exclude nonviable cells.

### Statistical analysis

The population for analysis was defined as randomly assigned patients eligible before treatment. Overall survival (OS) was defined as the time from random assignment to death from any cause. Recurrence-free survival (RFS) was defined as the time from randomization to confirmation of recurrence by the trial cancer board. Survival curves were estimated using the Kaplan–Meier method, and survival rates with 95% confidence intervals (CIs) were calculated. The survival rates between the treatment arms were compared using the log-rank test, and hazard ratios (HR) were calculated using the Cox proportional hazards model with and without the following covariates: age, sex, histology, stage, and preoperative chemotherapy. The significance level of the two-tailed statistical test was 0.05. Statistical analyses were performed using the Translational Research Informatics Center (TRI: TRILC1304) and by the Foundation for Biomedical Research and Innovation using SAS (version 9.3; SAS Institute, Cary, NC, USA). Interim analysis was scheduled for 5 years after the initiation of the study regardless of the number of enrolled patients.

## Results

### Consort diagram

**Fig. 1:**
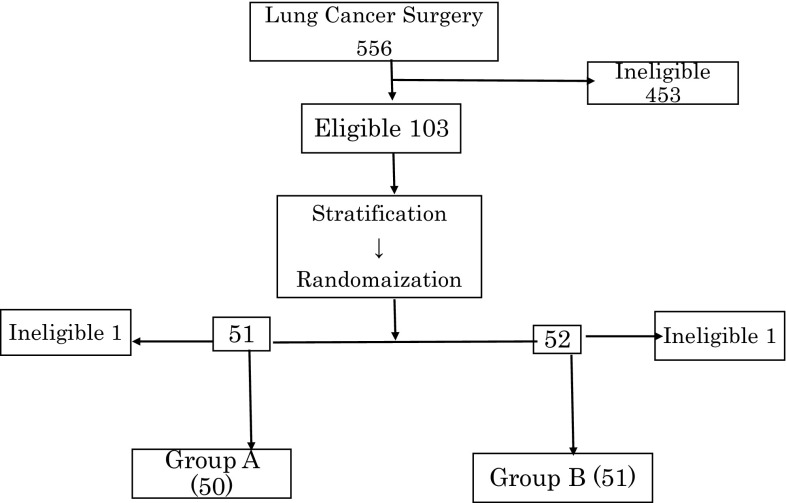
Consort diagram. Of the 556 patients who underwent surgery between April 2007 and July 2012, 103 were selected for randomization

As shown in Fig. 1, 453 of 556 patients who underwent surgery for NSCLC between April 2007 and July 2012 were excluded, and the remaining 103 patients were selected for randomization. Of the ineligible patients, 79 were excluded due to age (> 76 years), 303 were ineligible due to early-stage tumors. Among 62 patients with stage IIIB and IV cancer, a sufficient number of AKT–DC (> 7 × 10^9^) needed for a course of treatment could not be obtained because of immunosuppression in 35 cases (56.5%), and these patients were excluded from the study. Nine patients were excluded due to hepatitis viral infections or due to refusal to provide an informed consent for immunotherapy.

One patient each from groups A and B was excluded from the study due to a study violation or leukemic conversion of AKT–DC after randomization.

### Overall survival

**Fig. 2:**
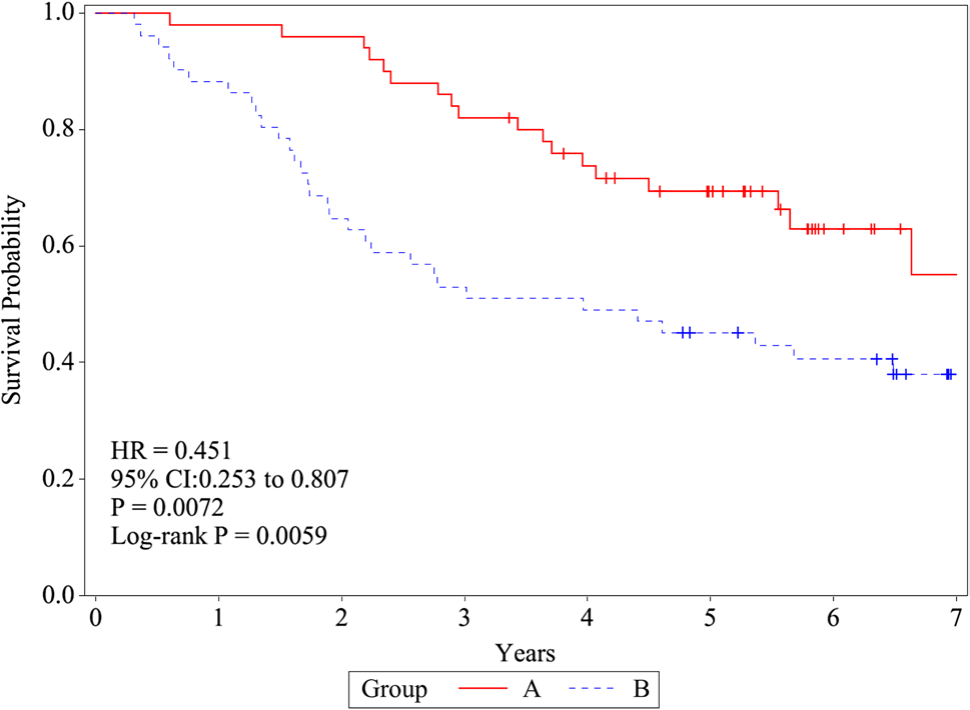
Overall survival (OS). OS was defined as the time from random assignment to death from any cause and was estimated using the Kaplan–Meier method

The difference in OS rates between the treatment arms was noted to be statistically significant (log-rank test, *P* = 0.0005) and in agreement with our initial findings from 4 years ago [6] (Fig. 2). The 2-, 5-, and 7-year OS rates were 96.0% (95% CI 84.9–99.0), 69.4% (54.4–80.3), and 55.1 (34.3–71.7) in group A and 64.7% (50.0–76.1), 45.1% (31.2–58.0), and 38.1% (24.6–51.4) in group B, respectively. The HRs were 0.451 (95% CI 0.253–0.807) by univariate and 0.439 (0.239–0.807) by multivariate analysis using treatment, age, sex, histology, stage, and preoperative chemotherapy.

### Recurrence-free survival

**Fig. 3:**
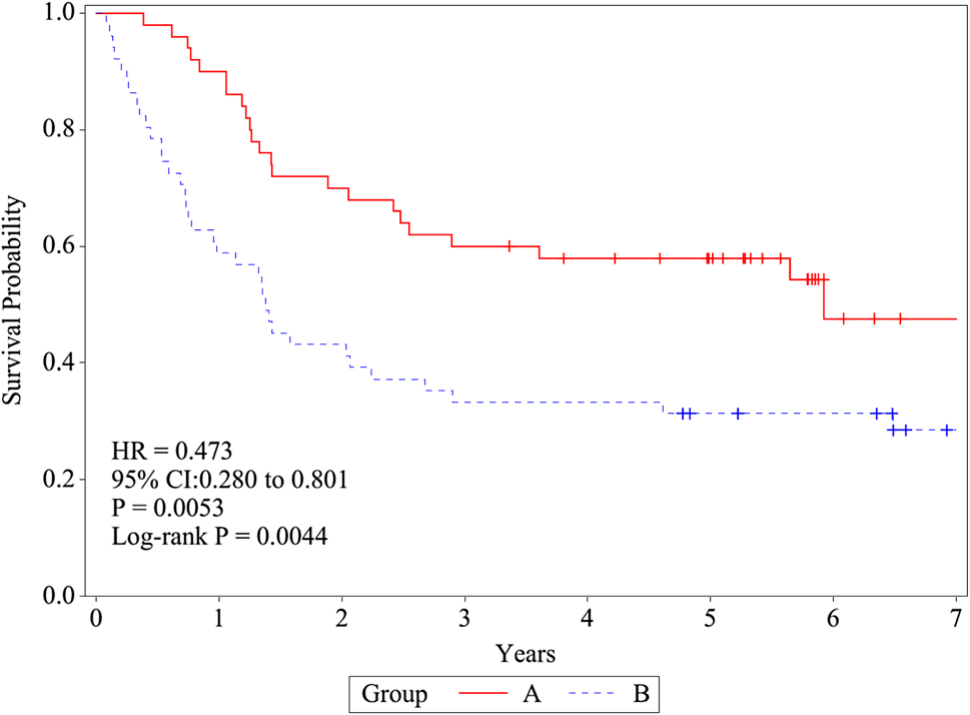
Recurrence-free survival (RFS). RFS was defined as the time from randomization to confirmation of recurrence by the trial cancer board. RFS was estimated using the Kaplan–Meier method

The 2-, 5-, and 7-year RFS rates were 70.0% (95% CI 55.3–80.7), 57.9% (43.1–70.2), and 47.5% (29.2–63.8) in group A and 43.1% (29.4–56.1), 31.4% (19.3–44.2), and 28.5% (16.7–41.5) in group B, respectively (Fig. 3). These differences in the RFS rates between the two treatment groups were also noted to be statistically significant (log-rank test, *P* = 0.0044). The HRs were 0.473 (95% CI 0.280–0.801) by univariate and 0.473 (0.275–0.812) by multivariate analysis in favor of group A.

### OS using Cox proportional hazards model for subgroup analyses and treatment interactions

The HRs by subgroup analysis of OS between treatment groups that were significantly lower than 1.0 in favor of the immunotherapy arm (Table 1) were as follows: 0.098 (0.011–0.856), age ≤ 55; 0.474 (0.248–0.907), males; 0.479 (0.239–0.959), adenocarcinoma; 0.399 (0.194–0.822), stage III tumors; and 0.483 (0.245–0.951), those without preoperative chemotherapy.

**Table 1:**
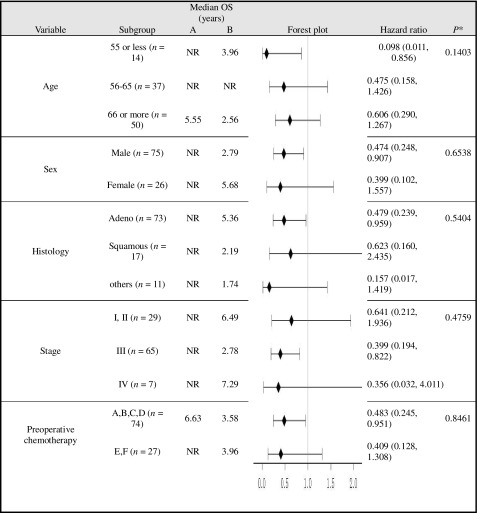
OS using Cox models for subgroup analyses and treatment interactions

*NR* not reached to 50%

*P value for treatment interaction

Platinum doublet regimens belonging to the third-generation drugs are used for an induction and adjuvant chemotherapy. Both groups received four courses of adjuvant chemotherapy after surgery (group a, b, c, and d). Stage IIIA patients (group e and f) received two courses of induction chemotherapy before surgery

### RFS using Cox proportional hazards model for subgroup analyses and treatment interactions

As shown in Table 2, the HRs (95% CIs) of RFS by subgroup analysis significantly lower than 1.0 in favor of the immunotherapy arm were as follows: 0.058 (0.007–0.520), age ≤ 55; 0.216 (0.059–0.791), females; 0.495 (0.269–0.909), adenocarcinoma; 0.446 (0.235–0.845), stage III tumors; and 0.507 (0.271–0.948), patients without preoperative chemotherapy (Table 2).

**Table 2:**
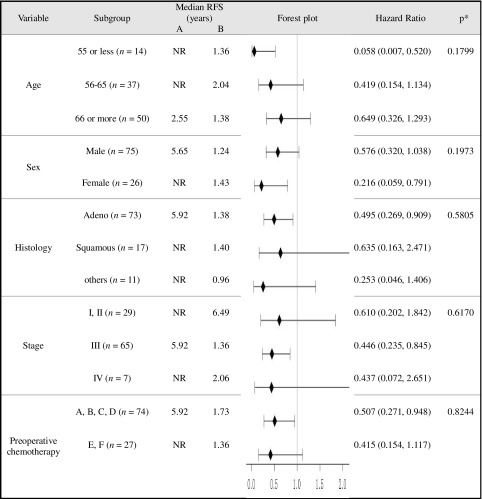
RFS using Cox models for subgroup analyses and treatment interactions

*NR* not reached to 50%

*P value for treatment interaction

### Cell surface markers and survival

**Fig. 4:**
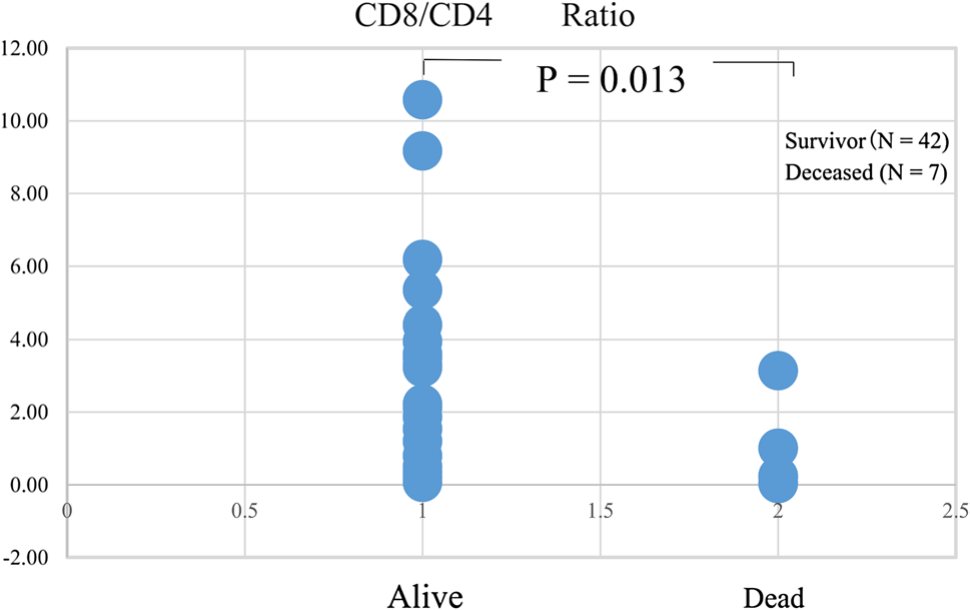
Cell surface markers and survival. The relationship between cell surface markers and survival was examined, which showed that the CD8^+^/CD4^+^ ratio was elevated in survivors

The CD8^+^/CD4^+^ T-cell ratio analyzed 1–2 months after the initiation of in vitro culture was higher in the survivors than in the deceased (*p* = 0.013: Fig. 4). Additional analyses using cell surface markers for determining the positive percentages of CD8^+^, CD4^+^, CD80^+^, CD83^+^, HLA-DR^+^, B7H1^+^, and T-reg (CD4 + CD25+) cells before and after in vitro culture failed to show significant relationships with survival.

## Discussion

This study is a series of adjuvant immunotherapy trials in patients with post-surgical lung cancer extending over 20 years, starting with Lymphokine activated killer (LAK) cells and continuing with AKT–DC. The first RCT conducted between 1986 and 1992 using LAK cells was reported in the journal *Cancer* [7]. The results of a phase II study conducted between 1998 and 2004 using AKT–DC obtained from the regional lymph nodes of patients with primary lung cancer predicted a promising outcome for a phase III study using this approach [8, 9]. The results of this phase III study clearly demonstrate that adoptive cellular immunotherapy benefits patients with lung cancer as an adjuvant to surgery.

Subgroup analysis of OS, comparing the immunotherapy arms using Cox models, indicated that younger patients, male patients, and patients with adenocarcinoma or stage III tumors are good candidates for immunotherapy. The prognosis for stages I and II is better than that for stage III in patients with NSCLC; however, chemoimmunotherapy improved prognosis for stage III more efficiently than it did for stage I and II; this finding was observed in both male and female patients. While the prognosis was better for females than for males, it was significantly improved by immunotherapy in males. Stage IIIB and IV tumors have evolved mechanisms for escaping immune response in the tumor microenvironment [10–13]. The immune system is either inefficient or tolerates the growth of tumors in stages IIIB or IV, which likely leads to ineffective chemoimmunotherapy outcomes in those patients. Lung cancer patients with stage I and II tumors are good candidates for surgery; however, surgery is not indicated in stage IIIA cases due to poor prognosis. If the prognosis of advanced NSCLC can be improved by cell-mediated immunotherapy, surgery may be added to the current treatment modalities for patients with stage IIIA cancer.

The assessment of histological types in this RCT showed that the HR of OS for adenocarcinoma was lower than that for squamous cell carcinoma. Patients with adenocarcinoma were demonstrated to benefit from immunotherapy. It is speculated that metastatic tumors resulting from circulating and disseminating tumor cells (CDTC), the primary residual adenocarcinoma constituents in these patients [14, 15], cannot escape immune surveillance and are eventually eliminated by cell-mediated immunotherapy [10, 16, 17]. Conversely, the residual pattern of squamous cell carcinoma includes residual edges of the resected primary tumor margin, but not CDTC. These residual tumors, like the original tumors, are capable of escaping immune surveillance blocking immune response. We excluded cases with macroscopically residual tumors; however, microscopically residual tumors, such as those with positive bronchial or chest wall margins, were included in the present trial. Squamous cell carcinoma invades the surrounding tissues that remain after resection, induces immunosuppression, and blocks immune responses, and may also prevent effective cell-mediated immunotherapy.

Patients who did not receive preoperative chemotherapy had lower HRs and benefited from immunotherapy, whereas patients who received preoperative chemotherapy did not significantly benefit from immunotherapy. Specific immune responses may be abrogated by preoperative chemotherapy. Cytotoxic anticancer drugs may negatively affect immune responses in regional lymph nodes, dampening the effect of immunotherapy.

Immunological analysis using cell surface markers of cultured lymphocytes indicated that the CD8^+^/CD4^+^ T-cell ratio was elevated in survivors. Analysis using other cell surface markers of lymphocytes before and after in vitro culture failed to show any significant correlation with survival. These results indicated that CD8^+^ cytotoxic T cells were more effective than CD4^+^ helper T cells in the adjuvant therapy circumstances in this study. The effect of direct cytotoxic killer T cells seems to be more significant than that of indirect support from helper T cells in eliminating CDTC.

Most cancer recurrences result from CDTC, which are clinically undetectable at the time of resection of a primary carcinoma [14, 15]. The immune response against CDTC released from primary tumors is distinct from that against original tumors regarding immunosuppression, which is induced by several immune escape mechanisms. Original tumors are not susceptible to cell-mediated immunotherapy due to several immune escape mechanisms, whereas CDTC may get eliminated by cell-mediated immunotherapy because they can evade neither immune surveillance nor immune response.

The target of immunotherapy in this trial was not the primary lesion, but the undetectable tumor cells remaining after the resection of primary carcinoma of the lung.

The phenotypic diversity of disseminated cells resulting from intra-tumor heterogeneity [18, 19] gives rise to clones that are resistant to chemotherapy and prevents tumor cell eradication by chemotherapy. The heterogeneity of tumor cells enables them to escape even from molecular-targeted therapy [20, 21]. The regional lymph nodes of patients with lung cancer are the organ where the first adaptive immune response against cancer develops [22, 23]. Dendritic cells at the tumor site internalize antigens, migrate to lymph nodes, and induce naive T cells to become antigen-specific cytotoxic T cells [24]. They act as messengers between the innate and adaptive immune response. From initiation to progression of cancer, tumor cells give rise to various antigens, which are recognized by dendritic cells. Regional lymph nodes represent one of the frontline defense mechanism against cancer to cope with heterogeneous cancer cells. Cell-mediated immunotherapy derived from regional lymph nodes as a source of dendritic cells and cytotoxic T cells may finally eradicate heterogeneous tumor cell clones that disseminate throughout the body, carrying a wide variety of antigens before immunosuppression develops in micrometastases. An important point to consider is whether the sufficient number of AKT–DC can be obtained from the patient for this cell-mediated immunotherapy, as we noticed that AKT–DC could not be obtained in the required quantities in nearly 56% of the stage IIIB and IV cases.

Even though our results suggest the clinical significance of cell-mediated immunotherapy along with chemotherapy for patients with lung cancer, there are certain limitations of this study. This study was carried out with a relatively small group of patients (only 103 patients) and at a single institution and only in Japan. Also, this was not a blinded study and the included patients were heterogeneous population. A large-scale, double-blind, randomized, multi-institutional trial is essential for ascertaining the efficacy of the presently described adjuvant cellular immunotherapy procedure and its clinical application. Successful dissemination of skills is required for the culture of regional lymph nodes to successors and this requires experience, time, and financial resources. Close cooperation and collaboration to extend the study protocol nationwide will be immensely beneficial to patients with lung cancer awaiting this cellular immunotherapy.

## Abbreviations

AKT–DC: Activated killer T cells and dendritic cells
CDTC: Circulating and disseminating tumor cells
CI: Confidence interval
HR: Hazard ratio
OS: Overall survival
RCT: Randomized controlled trial
RFS: Recurrence-free survival
